# Exploring the Psychosocial Factors between Adaptive and Maladaptive Use of Gaming among Korean Adolescents

**DOI:** 10.3390/children10061059

**Published:** 2023-06-14

**Authors:** Bee Kim, Nami Kim

**Affiliations:** Department of Addiction Science, Graduate School, Sahmyook University, Seoul 01795, Republic of Korea

**Keywords:** adolescent, adaptive game use, maladaptive game use, psychological factor, social factor

## Abstract

(1) Background: Various studies have been conducted on the effects of video (online, mobile, and console) games on users’ lives and psychological health. However, the effectiveness of a game can vary depending on user characteristics. This study explored the level of game use and its associated psychosocial factors among adolescents. (2) Methods: Survey data were compiled from 582 middle and high school students. Frequency analysis, the chi-square test, and analysis of variance were performed using SPSS Windows software, version 23.0. (3) Results: First, it was confirmed that there were no differences in the levels of self-esteem, morality, or life satisfaction between the adaptive game use and normal groups, but these were higher than those of the maladaptive game use group. However, at the level of self-control, the adaptive group scored lower than the normal group but higher than the maladaptive group. Second, the adaptive and normal groups exhibited comparatively lower levels of aggression, anxiety, depression, loneliness, academic stress, and social stress in school. Third, they also exhibit relatively high levels of social intelligence, social capital, and friendship support. (4) Conclusions: The adaptive and general game-use groups showed similar levels of psychosocial factors, whereas the maladaptive game-use group exhibited lower levels of positive psychological and social factors. Based on these results, developing an intervention program that reduces maladaptation and increases adaptive gaming use is necessary. Future follow-up studies are needed to confirm how positive and negative psychosocial factors affect adaptive and maladaptive game use as protective and risk factors, respectively.

## 1. Introduction

In Korea, in 2021, video (online, mobile, console) game users accounted for more than 70% of 10–65-year-olds. Furthermore, 93.7% of teenagers and 85.9% of 20-year-olds reported that they play games [1]. It was also found that 81.7% of parents played games with school-aged children [1]. Online games have become a common and essential part of daily life and are used not only for leisure but also as a venue for economic and social activities. During the recent U.S. presidential election, Joe Biden campaigned in the virtual reality of Nintendo’s *Animal Crossing* [2]. The K-pop group BTS (BangTan Sonyeundan or Beyond the Scene) released its new song, *Dynamite*, in the online game *Fortnite*, which was just like an actual concert site [3]. Games that were once considered “children’s play”, “simple leisure activities”, or were regarded as the cause of “diseases” or “pathological problems” if excessively used are now turning into a field of economic and social activities [4].

A meta-analysis conducted from 2013 to 2018 [5] investigating gaming addiction and its corresponding pathological classifications revealed a higher prevalence of research efforts concerning gaming addiction and maladaptive usage in Korea, compared to other nations. Furthermore, the majority of these studies operated under the assumptions of maladaptive usage or addiction, or from analogous perspectives. In particular, it targeted pedagogical and psychological effects on children and adolescents and focused on regulating youth game culture. These studies have focused mainly on individuals’ psychological and emotional states such as depression and anxiety, and problem behaviors such as aggression, addiction, and delinquency. These findings show that many studies in Korea highlighted games only as pathological problems, such as diseases, and were combined with social perceptions that only reinforced the negative aspects of games. 

However, research on the utility of positive or neutral games from an amusement perspective is worth discussing. An empirical study showed a difference between immersion and addiction in gaming and emphasized the distinction between using the Internet without pathological addiction and playing games as a dedicated enthusiast [6,7]. Moreover, a case study was conducted on having fun and leading a healthy daily life [8]. In addition, some studies discussed games from the positive psychological perspective that can positively affect adolescents [9,10], and others showed that games have a positive function as a learning tool [11]. A study found that games help relieve symptoms of depression or anxiety [12]. As such, even in previous studies on game use, there is a difference in the perspectives on adaptive and maladaptive game use.

Based on the previous studies that have classified game behavior types, rather than dividing the positive and problematic behaviors of game use, we explored each characteristic by combining the game use tendencies of the study participants. Lee’s (2007) study shows that even if online game usage is high, this does not necessarily lead to problematic game use, and it is believed that there is a stage of positive addiction in the process [13]. However, it is still necessary to be wary of the effect of immersion on problematic addiction when the motives and goals for game use are non-instrumental. This argument suggests that the impact of games varies based on individual characteristics and psychosocial factors. Therefore, it is necessary to focus on the game flow model, which acknowledges that the effects of games can differ based on individual psychosocial characteristics. 

Adaptive game use refers to the appropriate use of media for personal growth and development without negatively affecting health [14]. The Korea Creative Content Agency published a study showing that playing games has negative and positive effects on users [5]. In addition to games, some studies have shown that excessive use of smartphones has negative and positive effects [15,16,17]. These studies suggest that adaptative and maladaptive game use are continuous concepts and should be differentiated according to the “what kind of experience” one has rather than “use time”.

Previous studies on adaptive and maladaptive game use have shown that some people who play Internet games over a long period may develop pathological symptoms such as compulsive use and obsession, tolerance and withdrawal, dysfunction in daily life, deviant behavior, and reality discrimination disorder [18], while others may enjoy games and live healthy lives [8]. Therefore, rather than dividing the effects of games into positive and negative categories, they can be viewed as changing according to the characteristics of game users. 

Research on psychological and social factors associated with game use has been continuously reported. Self-control is important for predicting negative outcomes from problematic online game use [19,20,21]. Game addiction and self-esteem are negatively related [22], and a longitudinal study has confirmed that self-esteem is both a cause and consequence of pathological game use [23]. Positive correlations were observed between in-game and offline leadership [24], suggesting that leadership can be developed through in-game activities. It has been found that game use can have a positive moderating effect on loneliness [25]. Game use has been reported to generate positive effects such as satisfaction based on user needs, promotion of social relationships, and the possibility of being directly linked to life satisfaction or happiness [26]. 

Depression and anxiety significantly correlate with game addiction [27], as does aggression [20,28]. Adolescents would prefer interaction and achievement in the virtual world of Massively Multiplayer Online Role-Playing Games, where they can leave behind real-life flaws to interact with others [29]. Moreover, online social interactions have been shown to alleviate social isolation in older adults caused by reduced social roles and networks [30], and People in the virtual world engage in “social” activities and form relationships. Similarly, loneliness decreases when people receive support through social networking services activity [31]. This suggests that social interaction through games can reduce loneliness. 

Studies suggest that online gaming increases rather than hinders social capital [32,33]. Furthermore, social capital is stimulated by gamers’ relational desires, which, in turn, leads to the continued use of gaming [34]. In addition, gaming addiction can occur due to peer relationships [35]. During this period, gaming use is closely related to common interests, communication of thoughts and opinions, the level of support from peers [36], and The formation of social relationships, such as a sense of belonging and connection with peers, through gaming use has been reported to have a positive impact on adolescent life satisfaction [37]. 

Based on previous studies, this study examines the following research hypotheses: First, adaptive game use or general game use groups are likely to experience higher levels of positive psychological factors than those who exhibit maladaptive game use. Second, adaptive and general game-use groups will likely experience lower negative psychological factors than those with maladaptive game use. Third, adaptive and general game-use groups will likely experience higher social aspects than those with maladaptive game use. Therefore, this study aims to confirm the differences in positive, negative, and social psychological factors between adaptive and maladaptive game users.

## 2. Materials and Methods

### 2.1. Design, Setting, and Participants

In this study, ‘game’ as used in this study means a video game, and video games include computers, smartphone consoles, and arcades. Data from the “Game User Panel Survey” published by the Korea Creative Content Agency were used as secondary data after obtaining the necessary data authorization. The data used in the research analysis were 2018 data from the five-year period from 2014 to 2018 from a panel survey on game users. There were 287 middle school students (49.3%), 295 high school students (50.7%), 288 males (49.5%), and 294 females (50.5%), with 582 participants. 

### 2.2. Questionnaires

#### 2.2.1. Positive Psychological Factors

The Brief Self-Control Scale (BSCS) [38] was used to measure self-control (Mean (M) = 3.18, Standard Deviation (SD) = 0.55, and Cronbach’s α = 0.82). The Rosenberg Self-Esteem Scale (RSES) [39] was used to measure self-esteem (M = 2.05, SD = 0.50, Cronbach’s α = 0.87). The Altruism and Social Responsibility Scale of the Korea Youth Policy Institute [35] was used to measure morality (M = 2.84, SD = 0.28, and Cronbach’s α = 0.85). Leadership is rated on a five-point scale ranging from “1 = not at all” to “5 = very much so” with two items. “I often play the role of a student council executive or a leader in various gatherings (clubs, religious groups, etc.)” and “I usually give opinions, gather ideas, and lead actions among friends, and influence people regardless of status” (M = 3.14, SD = 0.98, and Cronbach’s α = 0.84). The Satisfaction with Life Scale (SWLS) was used to measure satisfaction with life [40] (M = 4.55, SD = 1.28, and Cronbach’s α = 0.94).

#### 2.2.2. Negative Psychological Factors

The Short-Form Buss–Perry Aggression Questionnaire (BPAQ-SF) [41] was used to measure aggression (M = 1.97, SD = 0.74, and Cronbach’s α = 0.93). The Generalized Anxiety Disorder (GAD-7) scale developed by Spitzer et al. [42] was used to measure anxiety (M = 0.46, SD = 0.61, and Cronbach’s α = 0.94). The Center for Epidemiological Studies Depression Scale (CESD)was used to measure depression (M = 0.39, SD = 0.45, and Cronbach’s α = 0.90). The UCLA (University of California, Los Angeles) Loneliness Scale [43,44] was used to measure the degree of loneliness (M = 1.62, SD = 0.60, and Cronbach’s α = 0.93). Among the life stress scales [45], four of ten academic factors and three of nine questions corresponding to friendship factors were selected to measure stress (M = 0.50, SD = 0.44, and Cronbach’s α = 0.80). 

#### 2.2.3. Social Factors

The Tromso-Social Intelligence Scale [46] measured social intelligence (M = 4.80, SD = 0.85, Cronbach’s α = 0.93). The Social Capital scale [47,48] was used to measure social capital (M = 3.71, SD = 0.59, and Cronbach’s α = 0.94). Three of the five questions on the scale [49] were selected (M = 3.55, SD = 0.86, and Cronbach’s α = 0.92) to measure teacher support. Among the teacher support scale [49], three out of five questions were selected, and they were rated on a four-point scale ranging from “1 = not at all” to “4 = always” (M = 3.20, SD = 0.58, and Cronbach’s α = 0.90).

#### 2.2.4. Adaptive Game Use

The adaptive game-use Scale [50] has the degree of vitality experience, life experience expansion, leisure use, immersion experience, pride experience, maintenance of social relationship support networks, and expansion experience obtained by users through games. Specifically, a total of 21 questions were asked, such as “Game brings life to life”, “Game gives me the energy to enjoy life”, “Game gives me the energy to talk about my problems”, “Game gives me energy”, and “Game gives me complete self-control”, which were all measured on a four-point scale ranging from “0 = not at all” to “3 = very much”. (Cronbach’s α = 0.97).

#### 2.2.5. Maladaptive Game Use

The Internet addiction scale [51] is reflected in in-game usage, and the measurement questions are “Have you ever played games for longer than you originally intended?”, “Have you ever played games before doing what you were supposed to do?”, “When you say ‘just a few more minutes’ while playing games, do you become engrossed in the game?”, “Have you ever felt excited about playing games again?”, “Has playing games caused problems in your school life?”, “Have you ever felt depressed and irritable when not playing games and then felt that all those feelings disappeared when you started playing games again?”, and “Do you prefer playing games over going outside with family and friends?” Responses were evaluated on a five-point scale ranging from “1 = little” to “5 = very much” (Cronbach’s α = 0.96).

### 2.3. Procedures and Data Analysis

In this study, the SPSS Windows software, version 23.0 (IBM, Armonk, NY, USA) was used as an analysis tool to estimate the correlation between variables. First, the adaptive and addictive game usage classification criteria were selected to classify the groups. For adaptive game use, if three or more of the seven sub-factors (scored 0–9 points) have a score 6 or higher, it is classified as “adaptive game user.” If the number of sub-factors with a score of 6 or higher is less than three, it is classified as a “non-adaptive game user” [50,52]. For an “addicted game user”, a score of 70 or higher is evaluated as an “addicted game user”, and scores lower than 69 are considered a “normal user” [51].

Based on the scale evaluation results, this study classified the participants into three groups to confirm the differences between the adaptive, maladaptive, and general game-use groups. General game users tend to see games as leisure or a hobby and usually experience minimal positive or negative effects from playing games. In contrast, adaptive game use involves the productive use of games for personal growth and development while avoiding any negative impact on one’s health by using them appropriately [14]. The maladaptive game use group included those with a high degree of problematic game use. 

The adaptive and normal game users were reclassified as the “adaptive game use group.” The problematic game users were reclassified as “maladaptive game use groups.” Non-adaptive and normal game users were reclassified into the “general game use group” for identification. The classification results were reviewed using discriminant analysis (classification accuracy: 81.4%), and one-way ANOVA was performed to determine whether there were differences in psychosocial factor levels between the groups. If the number of patients in each group was not equal, the difference between the groups was analyzed using Scheffé’s post hoc test.

## 3. Results

### 3.1. Socio-Demographic Characteristics of Respondents

To confirm the demographic characteristics of adolescents by game use level was calculated through a cross-analysis. The results are presented in <Table 1>. 

The analysis of the demographic characteristics according to the youths’ game use level showed that males (*n* = 41, 14.2%) in the maladaptive game use group showed approximately twice the rate of females (*n* = 20, 6.8%), which was similar to that in the adaptive game use group (males = 124, 43.1%; females = 78, 26.5%). The percentage of youths who reported playing Internet (online) games (*n* = 107, 46.1%) was higher than that of youths who reported playing mobile (cell phone) games or other games (PS2, PSP, arcade games, etc.). One youth group had a relatively high percentage of general users. In addition, it was confirmed that the ratio of the maladaptive game use group increased as game usage time increased.

### 3.2. Differences between Groups in the Positive Psychological Factors

A one-way ANOVA was performed to confirm the average difference in psychological factors by game use level among adolescents. This was calculated using the Scheffé post hoc test, and the results are presented in <Table 2>.

Looking at the difference in the positive psychological characteristics between the adolescent game use groups, the average self-control of the general user group (M = 3.30, SD = 0.56) was the highest. In the case of self-esteem, the averages of the general user group (M = 2.09, SD = 0.54) and adaptive game use group (M = 2.04, SD = 0.44) were higher than that of the maladaptive game use group (M = 1.90, SD = 0.47) (F = 3.80, *p* < 0.05). In the case of morality, the average of the maladaptive game use group (M = 2.64, SD = 0.35) was found to be the highest, followed by the average of the adaptive game use group (M = 2.52, SD = 0.23), and the general user group (M= 2.43, SD = 0.27) (F = 19.77, *p* < 0.001). In terms of life satisfaction, the averages of the general user group (M = 4.66, SD = 1.35) and the adaptive game use group (M = 4.50, SD = 1.19) were higher than that of the maladaptive game use group (M = 4.15, SD = 1.17) (F = 4.32, *p* < 0.05). Finally, there was no significant difference between the leadership groups (F = 2.48, *p* = 0.84). The maladaptive game-use group had the lowest average positive psychological factors, excluding leadership. The adaptive game-use group had a lower level of self-control than the general game-use group. However, self-esteem and life satisfaction were confirmed to be at the same level in the adaptive and general-use groups. Morality, which refers to altruism and social responsibility, was highest in the maladaptive game use group and higher in the adaptive game use than in the general use group.

### 3.3. Differences between Groups in the Negative Psychological Factors

A one-way ANOVA was performed to confirm the average difference in psychological factors by game use level among adolescents. This was calculated using the Scheffé post-hoc test, and the results are presented in <Table 3>.

When examining the variations in negative psychological characteristics among the adolescent game-use groups, the average aggressiveness of the maladaptive game-use group (M = 2.45, SD = 0.76) was the highest. The adaptive game use group (M = 2.08, SD = 0.68) scored higher than the general game-use group (M = 1.81, SD = 0.72) (F = 24.70, *p* < 0.001). Regarding anxiety, the average (M = 0.40, SD = 0.58) of the maladaptive game-use group was higher than that of the general game-use group (M = 0.57, SD = 0.52). However, the adaptive game-use group (M = 0.50, SD = 0.62) did not differ significantly (F = 4.08, *p* < 0.05). The average depression score in the maladaptive game use group (M = 0.57, SD = 0.52) was higher than that in the general use (M = 0.35, SD = 0.43) and maladaptive game use groups (M = 0.39, SD = 0.44) (F = 6.67, *p* < 0.01). The average loneliness of the maladaptive game use group (M = 2.01, SD = 0.64) was found to be the highest, followed by the adaptive game use group (M = 1.69, SD = 0.56) and the general user group (M = 1.50, SD = 0.57) (F = 22.39, *p* < 0.001). The average of the maladaptive game use group (M = 1.02, SD = 0.52) was the highest regarding academic stress. The average of the adaptive game use group (M = 0.77, SD = 0.46) was higher than that of the general use group (M = 0.63, SD = 0.46) (F = 19.39, *p* < 0.001). Finally, the maladaptive game use group (M = 0.40, SD = 0.56) was higher than the general (M = 0.23, SD = 0.38) and adaptive game use groups (M = 0.18, SD = 0.36) in terms of peer stress (F = 8.64, *p* < 0.001).

### 3.4. Differences between Groups in the Social Factors

A one-way ANOVA was performed to examine the mean difference in social factors by game-use level among adolescents. This was calculated using the Scheffé post hoc test, and the results are presented in <Table 4>.

When examining the difference in the social factors among the game use groups, the average social intelligence of the general uses group (M = 4.94, SD = 0.87) was the highest. The adaptive game use group (M = 4.73, SD = 0.76) scored higher than the maladaptive game use group (M = 4.27, SD = 0.75) (F = 17.74, *p* < 0.001). In the case of peer support, the averages of the general use group (M = 3.26, SD = 0.61) and the adaptive game use group (M = 3.14, SD = 0.53) were higher than that of the maladaptive game use group (M = 2.98, SD = 0.55) (F = 7.40, *p* < 0.01). Finally, there was no significant difference between groups regarding teacher support (F = 2.93, *p* = 0.54).

## 4. Discussion

Adaptive game use refers to positive psychological and behavioral experiences through gaming activities. This means experiencing vitality and pleasure through game activities and expanding thinking, immersion, competence, pride, maintenance, and expansion of social support networks, friendship, and self-control [53]. Such adaptive game use allows teenagers to use games efficiently for growth and development without negatively affecting their health. In particular, teenagers are now called “digital natives” [54], and peer-to-peer play culture is active through online games. Their lives are closely related to games, and the importance of using games has been emphasized. However, most of the previous studies have focused only on the negative effects of maladaptive game use, and discussions on the psychosocial factors of adaptive game use are insufficient. In this study, an analysis was conducted to examine the distinctions in psychosocial factor characteristics between the adaptive and maladaptive game-use groups. Furthermore, the research findings addressed these limitations and discussed the psychosocial factors associated with the adaptive game-use group. First, the adaptive game use group showed the same self-esteem, morality, and life satisfaction as the general user group. It showed higher self-control, self-esteem, morality, and life satisfaction than the maladaptive game-use group.

Conversely, the maladaptive game use group showed a lower level of self-control, self-esteem, morality, and life satisfaction than the adaptive and general game-use groups, indicating that the level of positive psychological factors in the maladaptive game user group was relatively lower than in the other groups. The level of self-control was the highest in the general game-use group, followed by the adaptive and maladaptive game use groups, which is also related to game usage time. These results were closely related to adaptive and maladaptive game use [21,55], and it was found that the lower the self-control, the more severe the maladaptive game use [56]. Additionally, Ko et al. [29] investigated the relationship between gaming and Internet addiction and found that low self-esteem among adolescents was reported as the cause of Internet addiction. A longitudinal study [23] showed that self-esteem was the cause of pathological game use and the result. However, the level of positive psychological factors, except for self-control, was the same, even though the adaptive game-use group had more time to use the game than the general game-use group. This is consistent with research results [16] that even if game usage is high, it does not necessarily lead to problematic game use. Even if the game capacity is higher than that of the general user group, it can be used adaptively. This means that even if the game use time is long, the game can be used well if the individual’s positive psychological factors are high. Previous studies have consistently shown that the adaptive game-use experience is positively related to self-esteem and subjective well-being [57,58]. Additionally, another study [26,59] reported that game use has the potential to enhance satisfaction, bonds of friendship, life satisfaction, and happiness. These findings collectively indicate that the game does not negatively affect individuals’ positive psychological factors. Instead, it has the potential to positively affect self-esteem and life satisfaction. 

Second, the adaptive game use group was similar to the general user group regarding anxiety, depression, and classmates’ stress. The levels of aggression, depression, loneliness, academic stress, and classmate stress were significantly lower in the adaptive game use group than in the maladaptive game-use group. These results support Baek et al.’s [37] findings that game use is positively related to peer relationships and academic achievement. The results of our study support those of other studies [31] in that loneliness is reduced when social activities are performed in a virtual world and social support is obtained. These results suggest that depressed individuals prefer online social interactions or relationship formation [60]. Nonetheless, adolescents who suffer from loneliness can avoid their own real-life deficiencies in the virtual world provided by Massively Multiplayer Online Role-Playing Games. It has been shown that exchange and a sense of achievement are the preferred interactions with real people [29]. This suggests that social interaction through games can be an alternative for reducing individuals’ feelings of depression, loneliness, and school and class stress. Although many previous studies have debated the effects of games on aggression [61,62], aggression can change according to the level of adaptive and maladaptive game use. It can be reduced or mitigated through game use [63]. This is due to the positive use of games such as improving problem-solving skills, relieving stress caused by the study, gaining vitality in life, and making new friends by using games through the characteristics of game use. Much effort is required to recognize and establish games as a healthy play culture, suggesting the need for psychosocial education to help young people perceive game experiences as positive. 

Third, the adaptive game use group showed the same level as the general use group regarding social capital and friend support and higher levels of social intelligence, social capital, and friend support than the maladaptive game use group. Social capital has recently attracted considerable attention from scholars among the various theories related to social networks. There has been a long debate over whether social capital formed in cyberspace, such as games, develops new social relationships or causes social fragmentation [64]. Putnam discussed the negative relationship between the Internet and social capital, stating that the Internet separates individuals from the local community and promotes personalization [65]. Nevertheless, in this study, no significant difference in social capital was found between the game- and general-use groups. In contrast, the maladaptive game-use group showed lower social capital than the adaptive and general user groups. This study showed that the Internet increases social capital rather than hindering it [32,33,66,67] and connects friends and relatives. Contrary to studies claiming to increase social capital by playing a role [68], the maladaptive use of games negatively affects social capital. Moreover, it is essential to use the game well. 

Social intelligence also confirmed the difference between adaptive and maladaptive user groups. This difference is attributed to the adaptive use group receiving positive psychological experiences through games—learning about society and culture and improving social intelligence through the expansion of thinking—compared with the maladaptive use group. In addition, because social intelligence refers to “the ability to understand social cues and act wisely in social and interpersonal relationships” [46], adolescents’ social intelligence plays an important role in friendships. Game use during this period was closely related to the level of communication and support for common interests, thoughts, and opinions among peers. Although helpful [37], when people become immersed in games, they depend on peer relationships online rather than playing or taking advantage of games [69]. This tendency indicates a relatively low level of support for peers compared with the well-used group by providing limited experience in support for peers. Game use during this period was closely related to the level of communication and support for common interests, thoughts, and opinions among peers. The formation of social relationships, such as a sense of connection and belonging to classmates, obtained through game use is helpful for life satisfaction in adolescents [37]. However, when individuals become maladaptive users, they depend on peer relationships online rather than playing games or taking advantage of them [69]. This tendency indicates a relatively low level of support for peers compared to the adaptive use group because it provides a limited experience of support for peers. However, adaptive game use can be an important means and space to expand social relationships and can be evaluated and expressed by adolescents [70]. Therefore, social support can be received, positively affecting an individual’s psychological development. 

This study had some limitations. First, based on the level of gaming time and psychosocial factors, adaptive game use may be a precursor to maladaptive game use. In future research, it will be necessary to examine whether adaptive game use groups develop into maladaptive game use groups over time, depending on differences in game usage time and psychosocial factors.

The game addiction group consisted of individuals with high game immersion and usage levels. Therefore, there are limitations to separating the two groups into independent and mutually exclusive groups, as the addiction group includes the characteristics of the usage group. In future research, a clear premise is required to determine whether to construct independent, sequential, or simply coexisting relationships between groups to clarify the differences and characteristics of groups’ in-game usage.

Third, this study failed to consolidate the individual psychosocial differences arising from game usage, such as overuse or excessive use, into a single theoretical framework. Although no differences were found between the addiction and general usage groups at specific levels of psychosocial factors, this is considered a characteristic of each factor. Emphasis on the importance of positive psychological factors can still be used as empirical evidence for a positive psychological approach.

Finally, the psychosocial factors measured in this study were self-reported Likert scales that focused on the relationship between game-use behavior and factors. Therefore, a comprehensive approach to how the quality of factors affects game-use behavior is limited in scope.

## 5. Conclusions

This study provides insights into the psychological and social factors associated with adaptive and maladaptive game use. The findings suggest that the maladaptive game-use group exhibited lower levels of positive psychological and social factors. In contrast, the adaptive and general user groups showed similar psychosocial factors. Therefore, intervention programs targeting maladaptive game use are important to reduce maladaptive game use through adaptive gaming gradually. This study provides a foundation for developing such programs and highlights the need for further research on the positive effects of adaptive game use on individuals’ psychosocial levels.

Additionally, this study indicated that game use time alone may not be a reliable predictor of psychosocial factors. While the maladaptive game use group showed the longest game use time, the adaptive game use group had a longer time than the general user group but showed similar psychosocial factors. Future studies should explore other factors influencing the relationship between gaming use and psychosocial outcomes. 

Overall, this study underscores the importance of addressing maladaptive game use through targeted interventions and provides insights into the complex interplay between game use and psychosocial factors. In promoting the adaptive use of gaming, promoting a healthy gaming experience is crucial by going beyond eliminating negative factors and providing education that enhances positive psychological and social strengths. Simultaneously, games literacy education should be promoted to acquire a healthy approach to game use. It is also possible to turn students’ experiences in the online world through gaming into career education that they can connect to their future careers. This will foster healthy game use and contribute to the prevention of negative outcomes, necessitating the development of effective strategies.

## Figures and Tables

**Table 1:** Demographic characteristics of the game use groups.

Variables		Game Use Groups				
		General User (*n* = 319)	Adaptive User (*n* = 202)	Maladaptive User (*n* = 61)	Total (*n* = 582)	X^2^
Sex	Male	123 (38.6%)	124 (61.4%)	41 (67.2%)	288 (49.5%)	34.35 **
	Female	196 (61.4%)	78 (38.6%)	20 (32.8%)	294 (50.5%)	
Grade	Middle 2nd	152 (47.6%)	98 (48.5%)	37 (60.7%)	287 (49.3%)	3.54
	High 2nd	167 (52.4%)	104 (51.5%)	24 (39.3%)	295 (50.7%)	
The most played game	Internet (online) game	94 (29.5%)	107 (53.0%)	31 (50.8%)	232 (39.9%)	37.89 **
	Mobile game	183 (57.4%)	88 (43.6%)	24 (39.3%)	295 (50.7%)	
	Others (PS2: PlayStaion 2, PSP: PlayStation Portable, etc.)	42 (13.2.%)	7 (3.5.%)	6 (9.8%)	55 (9.5%)	
Gaming hours	Less than an hour	173 (54.2%)	52 (25.7%)	8 (13.1%)	233 (40.0%)	84.27 **
	1–2 h	68 (21.3%)	54 (26.7%)	14 (23.0%)	136 (23.4%)	
	2–3 h	43 (13.5%)	50 (24.8%)	14 (23.0%)	107 (18.4%)	
	3–4 h	23 (7.2%)	27 (13.4%)	10 (16.4%)	60 (10.3%)	
	More than 4 h	12 (3.8%)	19 (9.4%)	15 (24.6%)	46 (7.9%)	

** *p* < 0.01.

**Table 2:** Differences in the positive psychological factors between the groups.

Variables	Groups	Mean	SD	F(df)	Scheffe
Leadership	General (*n* = 319)	3.17	1.05		-
	Adaptive (*n* = 202)	3.18	0.90	2.48 (2579)	
	Maladaptive (*n* = 61)	2.88	0.91		
Self-control	General	3.30	0.56		3 < 2 < 1
	Adaptive	3.10	0.48	24.85 (2579) **	
	Maladaptive	2.83	0.47		
Self-efficacy	General	2.09	0.54		3 < 1·2
	Adaptive	2.04	0.44	3.80 (2579) *	
	Maladaptive	1.90	0.47		
Morality	General	2.89	0.37		3 < 1·2
	Adaptive	2.82	0.36	19.43 (2579) **	
	Maladaptive	2.57	0.32		
Satisfaction with life	General	4.66	1.35		3 < 1·2
	Adaptive	4.50	1.19	4.32 (2579) *	
	Maladaptive	4.15	1.17		

* *p* < 0.05 ** *p* < 0.01. 1 = general group; 2 = adaptive group; 3 = maladaptive group.

**Table 3:** Differences in the negative psychological factors between groups.

Variables	Groups	Mean	SD	F(df)	Scheffe
Aggression	General (*n* = 319)	1.81	0.72		1 < 2 < 3
	Adaptive (*n* = 202)	2.08	0.68	24.70 (2579) **	
	Maladaptive (*n* = 61)	2.45	0.76		
Anxiety	General	0.40	0.58		1 < 3
	Adaptive	0.50	0.62	4.08 (2579) *	
	Maladaptive	0.62	0.67		
Depression	General	0.35	0.43		1·2 < 3
	Adaptive	0.39	0.44	6.67 (2579) **	
	Maladaptive	0.57	0.52		
Loneliness	General	1.50	0.57		1 < 2 < 3
	Adaptive	1.69	0.56	22.39 (2579) **	
	Maladaptive	2.01	0.64		
Academic stress	General	0.63	0.46		1 < 2 < 3
	Adaptive	0.77	0.46	19.39 (2579) **	
	Maladaptive	1.02	0.52		
Peer stress	General	0.18	0.36		1·2 < 3
	Adaptive	0.23	0.38	8.64 (2579) **	
	Maladaptive	0.40	0.56		

* *p* < 0.05 ** *p* < 0.01. 1 = general group; 2 = adaptive group; 3 = maladaptive group.

**Table 4:** Differences in the social factors between the groups.

Variables	Groups	Mean	SD	F(df)	Scheffe
Social intelligence	General (*n* = 319)	4.94	0.87		3 < 2 < 1
	Adaptive (*n* = 202)	4.73	0.76	17.74 (2579) **	
	Maladaptive (*n* = 61)	4.27	0.75		
Social capital	General	3.75	0.62		3 < 1·2
	Adaptive	3.68	0.55	3.87 (2579) **	
	Maladaptive	3.53	0.51		
Teacher support	General	3.61	0.91		-
	Adaptive	3.52	0.82	2.93 (2579)	
	Maladaptive	3.33	0.66		
Peer support	General	3.26	0.61		3 < 1·2
	Adaptive	3.14	0.53	7.40 (2579) **	
	Maladaptive	2.98	0.55		

** *p* < 0.01. 1 = general group; 2 = adaptive group; 3 = maladaptive group.

## Data Availability

www.kocca.kr/gameguide/subPage.do?menuNo=203709.

## References

[1] KOCCA (2021). 2021 Game Users Survey.

[2] Ko E.K. In ‘Nintendo Animal Crossing’, Biden of the US Laughed and Ishiba of Japan Cried. https://www.hankookilbo.com/News/Read/A2020090911540002114.

[3] Kim J.S. BTS Universe Story Can Be Changed by Your Choice. https://www.thisisgame.com/webzine/nboard/5/?n=109554.

[4] Kim N.R. Metaverse Alliance Launched. https://www.asiatoday.co.kr/view.php?key=20210727010015635.

[5] KOCCA (2018). Complementary Research on the Comprehensive Diagnostic Scale for Game Behavior (CSG).

[6] Lee S.C., Kim N.H., Suh Y.H. (2003). The Effect of Flow and Addiction upon Satisfaction and Customer Loyalty in Online Games. Korean Manag. Rev..

[7] Kwon J.W. (2004). A qualitative Case Study about Effect of Game-Type Substitution Therapy on Adolescent’s Game Addiction Treatment. Korean J. Youth Stud..

[8] Yoo S.H., Chong U.C. (2001). A Study on the Effects of Game Use on Adolescents. Natl. Youth Policy Inst..

[9] Csikszentmihalyi M. (1975). Beyond Boredom and Anxiety.

[10] Seligman M.E., Csikszentmihalyi M. (2014). Positive psychology: An introduction. Flow and the Foundations of Positive Psychology.

[11] Al-Azawi R., Al-Faliti F., Al-Blushi M. (2016). Educational gamification vs. game based learning: Comparative study. Int. J. Innov. Manag. Technol..

[12] Jeong Y.K. “Overcoming Depression through Games”… A Game That Evolves from Play to ‘Cure’. https://www.news1.kr/articles/?4171705.

[13] Lee S.K. (2007). Positive Addiction of Computer(On-line) Games. J. Cybercommunication Acad. Soc..

[14] Ko S.J., Park S.D., Hong S.K., Lee S.H., Lee H.Y. (2018). The Development and the Validity of Evaluation Scale for the Good Use of Smartphone for Middle School Students. J. Youth Welf..

[15] Israelashvili M., Kim T., Bukobza G. (2012). Adolescents’ over-use of the cyber world–Internet addiction or identity exploration?. J. Adolesc..

[16] Livingstone S. (2003). Children’s use of the internet: Reflections on the emerging research agenda. New Media Soc..

[17] Tzavela E.C., Karakitsou C., Dreier M., Mavromati F., Wölfling K., Halapi E., Macarie G., Wójcik S., Veldhuis L., Tsitsika A.K. (2015). Processes discriminating adaptive and maladaptive Internet use among European adolescents highly engaged online. J. Adolesc..

[18] Kim C.T., Kim D.I., Park J.G., Lee S.J. (2003). Internet Addicted Persons Diagnostic Test (K-Scale) and Preventive Education Program.

[19] Kim S.H., Hwang S.H. (2020). Effects of Adolescents’ Game Addiction on Depression: Moderating Effect of Self-control. J. Korea Converg. Soc..

[20] Jang Y.B. (2017). Effects of Loneliness, Aggression, Self-Control and Morality on Game Over-Indulgence Among Adolescents. J. Korean Soc. Comput. Game.

[21] Haagsma M.C., Caplan S.E., Peters O., Pieterse M.E. (2013). A cognitive-behavioral model of problematic online gaming in adolescents aged 12–22 years. Comput. Hum. Behav..

[22] Lee Y.J., Park M.K. (2020). The Effect of Game Addiction on Subjective Health Status of Out-of-School Youths: The Mediating Effects of Self-Esteem and Social Stigma. Korean Soc. Wellness.

[23] Lemmens J.S., Valkenburg P.M., Peter J. (2011). Psychosocial causes and consequences of pathological gaming. Comput. Hum. Behav..

[24] Jang Y.B., Ryu S.H. (2010). Exploring game experiences and game leadership in massively multiplayer online role-playing games. Br. J. Educ. Technol..

[25] Lee D.Y., Lee S.J., Jeong Y.J. (2016). Effect of Adolescent’s Game Using Time on Morality and Psychological Factors. J. Korean Soc. Comput. Game.

[26] Lee H.R., Jeong E.J. (2015). The Influence of Players’ Self-Esteem, Game-Efficacy, and Social Capital on Life Satisfaction Focused on Hedonic and Eu-daimonic Happiness. J. Korea Multimed. Soc..

[27] Kim M.J. (2018). The Pathways from Family Relationship Quality, Adolescents’ Depression and Anxiety to Game Addiction-Focusing on Gender Differences. Korean Soc. Wellness.

[28] Mehroof M., Griffiths M.D. (2010). Online gaming addiction: The role of sensation seeking, self-control, neuroticism, aggression, state anxiety, and trait anxiety. Cyberpsychol. Behav. Soc. Netw..

[29] Ko C.H., Yen J.Y., Chen C.C., Chen S.H., Yen C.F. (2005). Gender differences and related factors affecting online gaming addiction among Taiwanese adolescents. J. Nerv. Ment. Dis..

[30] Lee H.J., Lim J.S. (2019). Influence of Social Isolation on Smart Phone Addiction through Self-regulation and Social Support. J. Korea Contents Assoc..

[31] Son Y.J., Heo M.S. (2020). A Study on Social Media Usage, Helplessness, and Loneliness Experienced by College Students since the COVID-19 Pandemic. J. Korea Contents Assoc..

[32] Kobayashi T. (2010). Bridging social capital in online communities: Heterogeneity and social tolerance of online game players in Japan. Hum. Commun. Res..

[33] Cole H., Griffiths M.D. (2007). Social interactions in massively multiplayer online role-playing gamers. Cyberpsychol. Behav..

[34] Chae S.W., Kang Y.J. (2016). The Effect of Mobile Network Social Gamers’ Altruism on Continuous Usage Intention: The Mediating Effect of Social Relational Capital. J. Inf. Syst..

[35] Kim K.J., Oh H.S., Jung I.J., Oh M.S. (2011). Youth Mentoring Pilot Project and Measurement of Its Effects.

[36] Xu X., Zhao Y.C., Zhu Q. The Effect of Social Media Use on Older Adults’ Loneliness-The Moderating Role of Self-disclosure. Proceedings of the International Conference on Human-Computer Interaction.

[37] Baek K., Yoo M., Kang H., Cho M. (2020). The influence of social relationship on adaptive and maladaptive game use. J. Korea Contents Assoc..

[38] Tangney J.P., Boone A.L., Baumeister R.F. (2018). High self-control predicts good adjustment, less pathology, better grades, and interpersonal success. Self-Regulation and Self-Control.

[39] Rosenberg M. (2015). Society and the Adolescent Self-Image.

[40] Lim N.Y., Lee H.R., Seo E.K. (2010). Overview of Diener’s Satisfaction with Life Scale (SWLS) in Korea. Korean J. Psychol. Gen..

[41] Diamond P.M., Wang E.W., Buffington-Vollum J. (2005). Factor structure of the Buss-Perry Aggression Questionnaire (BPAQ) with mentally ill male prisoners. Crim. Justice Behav..

[42] Spitzer R.L., Kroenke K., Williams J.B., Löwe B. (2006). A brief measure for assessing generalized anxiety disorder: The GAD-7. Arch. Intern. Med..

[43] Knight R.G., Chisholm B.J., Marsh N.V., Godfrey H.P. (1988). Some normative, reliability, and factor analytic data for the revised UCLA Loneliness Scale. J. Clin. Psychol..

[44] Miller T.R., Cleary T.A. (1993). Direction of wording effects in balanced scales. Educ. Psychol. Meas..

[45] Kim K.H., Jeon G.G. (1993). Development and study of life stress and coping scale for middle school students. Korean J. Clin. Psychol..

[46] Silvera D., Martinussen M., Dahl T.I. (2001). The Tromsø Social Intelligence Scale, a self-report measure of social intelligence. Scand. J. Psychol..

[47] Putnam R.D. (1996). The strange disappearance of civic America. Policy J. Public Policy Ideas.

[48] Bourdieu P. (2011). The forms of capital (1986). Cult. Theory Anthol..

[49] Lee K.J. (1997). Stress, Social Support, and Behavior Problems of Adolescents. Doctor’s Dissertation.

[50] KOCCA (2010). Comprehensive Scale for Assessing Game Behavior Manual.

[51] Young K.S. (2009). Internet Addiction Test.

[52] KOCCA (2017). A First-Year Study of the Game User Panel.

[53] KOCCA (2021). Development of Psychosocial Model for Gaming Overindulgence Intervention.

[54] Prensky M. (2001). The games generations: How learners have changed. Digit. Game-Based Learn..

[55] Khang H., Kim J.K., Kim Y. (2013). Self-traits and motivations as antecedents of digital media flow and addiction: The Internet, mobile phones, and video games. Comput. Hum. Behav..

[56] Safarina N., Halimah L. (2019). Self-Control and Online Game Addiction in Early Adult Gamers. J. Phys. Conf. Ser..

[57] Jang H. (2001). The Relationship between Flow and Psychological Adjustment among Internet User. Master’s Thesis.

[58] Kim J.W., Lee H.R., Jeong E.J. (2015). The Effect of Game Players Personality, Social Capital and Adaptive Game Use on Life Satisfaction. Korean Soc. Comput. Game.

[59] Kim I.H. (2018). The Differences of Game Flow and Game Addiction: Focusing on Self-Efficacy, Life-Satisfaction, Interpersonal Problems. Master’s Thesis.

[60] Caplan S.E. (2003). Preference for online social interaction: A theory of problematic Internet use and psychosocial well-being. Commun. Res..

[61] Olson C.K. (2004). Media violence research and youth violence data: Why do they conflict?. Acad. Psychiatry.

[62] Markey P.M., Scherer K. (2009). An examination of psychoticism and motion capture controls as moderators of the effects of violent video games. Comput. Hum. Behav..

[63] Quwaider M., Alabed A., Duwairi R. (2019). The impact of video games on the players behaviors: A survey. Procedia Comput. Sci..

[64] Lee H.K. (2003). Effects of Individual- and Social-related Factors and Motives for Game Playing on Game Concentration and Game Addiction. Korean J. Youth Stud..

[65] Putnam R.D. (1995). Tuning in, tuning out: The strange disappearance of social capital in America. PS Political Sci. Politics.

[66] Williams D., Ducheneaut N., Xiong L., Zhang Y., Yee N., Nickell E. (2006). From tree house to barracks: The social life of guilds in World of Warcraft. Games Cult..

[67] Steinkuehler C.A., Williams D. (2006). Where everybody knows your (screen) name: Online games as “third places”. J. Comput.-Mediat. Commun..

[68] Quan-Haase A., Wellman B. (2004). Local virtuality in a high-tech networked organization. Anal. Krit..

[69] Collins E., Freeman J. (2013). Do problematic and non-problematic video game players differ in extraversion, trait empathy, social capital and prosocial tendencies?. Comput. Hum. Behav..

[70] Cho N.A. (2012). The Study about Social Media Literacy of Youths. Korean J. Youth Welf..

